# Identifying Biopsychosocial Characteristics of Emergency Youth Shelter Residents With Psychiatric Diagnoses

**DOI:** 10.7759/cureus.45355

**Published:** 2023-09-16

**Authors:** Dao Le, Caroline E Kelmis, Andrew J Ferdock, Amy B Smith, Hoonani M Cuadrado, Marna R Greenberg

**Affiliations:** 1 Department of Emergency and Hospital Medicine, Lehigh Valley Health Network/USF (University of South Florida) Morsani College of Medicine, Allentown, USA; 2 Department of Education, Lehigh Valley Health Network/USF (University of South Florida) Morsani College of Medicine, Allentown, USA; 3 Department of Street Medicine, Lehigh Valley Health Network/USF (University of South Florida) Morsani College of Medicine, Allentown, USA

**Keywords:** homeless, homeless youth, suicide risk, biopsychosocial, mental disorders, adolescent, youth

## Abstract

Background

In the United States, homelessness is an issue that may affect a significant portion of the adolescent population. There is no consensus on the extent to which this population has been impacted by poor mental health and lack of resources. This study aimed to characterize trends among those who struggle with housing insecurity and mental illness to provide a clearer picture of mental health needs among this population.

Methods

Data from 641 adolescents who presented to a local adolescent homeless shelter between 2015 and 2021 were utilized to determine if there were significant associations between specific mental illness diagnoses and biopsychosocial characteristics. A chi-square test of independence was performed on demographic and psychosocial variables for categories with a frequency greater than five. For continuous variables, an unpaired t-test was utilized to assess significance (p<0.05).

Results

Among the study population, 61.3% (369) had at least one psychiatric diagnosis, which is higher than even the most conservative estimates of mental illness among the general public. Having one or more psychiatric diagnoses was significantly associated with suicide attempts, documented aggressive behavior, and tobacco use. Contrary to our initial hypothesis, there were no significant correlations between psychiatric diagnoses and demographic characteristics or drug use other than tobacco.

Conclusions

Our findings indicate that though the particular reasons for homelessness among adolescents may vary, the prevalence of mental illness among these young individuals was roughly uniformly distributed and vastly above normal levels. Future research must focus on developing interventions to mitigate the effects of mental illness among homeless adolescents, as they are at a vulnerable point in their formative years.

## Introduction

Between 2016 and 2017, one out of every 30 adolescents experienced homelessness in the United States [1]. Unstably housed adolescents (homeless and runaway youth) have been shown to have inferior mental health compared to stably housed individuals of the same age group, with a much higher likelihood of suicide attempts [2]. Though these discrepancies in mental health outcomes are apparent, access to mental health care for homeless youth remains limited, with adolescent suicide continuing to become a larger public health issue [3]. Greater efforts must, therefore, be made to improve mental health outcomes for homeless adolescents. To better combat adolescent suicide and poor mental health outcomes, it is imperative to understand the conditions that breed such poor outcomes.

In 2015, a clinic was established at Valley Youth House, a shelter for adolescents experiencing homelessness in southeastern Pennsylvania, to ensure that the population in this area could have more secure access to medical and psychiatric care. A previous analysis showed that 54.9% of residents receiving services from the Valley Youth House clinic had a diagnosed psychiatric illness [4]. The purpose of this study was to determine psychiatric illness burden and biopsychosocial factors correlated with psychiatric illness in the homeless adolescent population, including gender, race, ethnicity, histories of suicide attempts, aggressive behavior, traumatic events, substance use, and risky sexual activity among homeless adolescents.

## Materials and methods

An IRB-approved retrospective chart review study was performed using data gathered during state-mandated physicals at Valley Youth House from 2015 to 2021. Charts were extracted by the study team including demographic information, childhood trauma and abuse history, substance abuse history, medical and psychiatric diagnoses, and sexual activity. These data were self-reported by the youth at the shelter during their physicals. Only charts from each adolescent’s initial encounter were used. Thus, if a person returned to the shelter and clinic more than once, subsequent assessments were excluded. A total of 602 charts were included in this study. Participant ages ranged between 11 and 20 years. Only the frequency of the primary psychiatric diagnosis, the first diagnosis listed on the chart and the one with more predominant symptoms for the youth, was calculated.

Descriptive statistics were used to assess the demographic characteristics and baseline behavioral, social, and sexual history of the total sample, as well as adolescents with and without psychiatric diagnoses. The mean and standard deviation were calculated for normally distributed continuous variables. Frequencies and percentages were used to describe categorical variables. To test the hypothesis that certain demographic and biopsychosocial characteristics were correlated with a psychiatric diagnosis, a chi-square test of independence was performed for categories with a frequency greater than five. For continuous variables, an unpaired t-test was utilized to assess for significance. A p-value below 0.05 was considered statistically significant.

We hypothesized that males and females would be equally affected by psychiatric diagnoses, with a larger disease burden on transgender and nonbinary populations. We also predicted that non-White and Hispanic populations would have a greater disease burden of psychiatric illness and a positive correlation between psychiatric diagnoses with suicide attempts, aggressive behavior, traumatic events, substance use, and risky sexual activity in comparison to the cohort without psychiatric diagnoses.

## Results

In total, 602 charts met the inclusion criteria of having been performed during an adolescent’s first visit to the clinic. Thirty-nine charts were completed during non-index visits and were excluded from analyses due to redundancy. Only 331 of the 602 charts contained information on race and psychiatric diagnoses, while only 269 of the charts contained data on ethnicity. A majority of adolescents who presented to Valley Youth House were female (n = 343, 57.00%), White (n = 162, 48.94%), and Hispanic/Latino (n = 148, 55.02%) with a mean age of 15.40 (SD = 1.64). Of the 602 charts used for analysis, four were missing information on current or previous psychiatric diagnoses. Three hundred and sixty-nine adolescents (61.30%) had a record of at least one psychiatric diagnosis. Demographics are further described in Table 1. A history of psychiatric diagnoses was not significantly correlated with age, gender, race, or ethnicity.

**Table 1:** Demographics and Differences Between Adolescents in This Study Population With and Without Psychiatric Diagnoses. ^a^The p-value was used to compare those with at least one psychiatric diagnosis and those without. The unpaired t-test was used for age, and the chi-square test was used for all other variables, excluding categories with counts less than five. ^b^Two hundred and seventy records were excluded for missing race data. Race was self-reported and some adolescents reported their own race as being Hispanic/Latino. One record was excluded for missing psychiatric diagnosis data. ^c^Three hundred and thirty-three records were excluded for missing ethnicity data.

Demographics	Entire Sample (N = 602)	Presence of Psychiatric Diagnosis (N = 369)	No Psychiatric Diagnosis (N = 229)	p-Value^a^
Age Mean (SD)	15.40 (1.64)	15.44 (1.68)	15.33 (1.58)	0.4205
Gender n (%)				
Male	245 (40.70)	144 (39.02)	99 (43.23)	0.3924
Female	343 (57.00)	214 (57.99)	127 (55.46)	
Transgender	10 (1.66)	7 (1.90)	3 (1.31)	
Other	4 (0.66)	4 (1.08)	0	
Race^b^ n (%)	(N = 331)	(N = 229)	(N = 102)	0.1586
Black/African-American	97 (29.30)	62 (27.07)	35 (34.31)	
White	162 (48.94)	121 (52.84)	41 (40.20)	
Asian	6 (1.81)	4 (1.75)	2 (1.96)	
Hispanic/Latino	16 (4.83)	11 (4.80)	5 (4.90)	
American Indian/Alaskan Native	0	0	0	
Other	3 (0.90)	2 (0.87)	1 (0.98)	
Multiracial	47 (14.20)	29 (12.66)	18 (17.65)	
Ethnicity^c^ n (%)	(N = 269)	(N = 192)	(N = 77)	0.8631
Hispanic/Latino	148 (55.02)	105 (54.69)	43 (55.84)	
Not Hispanic/Latino	121 (44.98)	87 (45.31)	34 (44.16)	

Regarding primary psychiatric diagnosis, the highest frequency of adolescents (n = 176, 47.70%) reported a diagnosis of depression (Table 2).

**Table 2:** Counts of Homeless Adolescents’ Primary Psychiatric Diagnoses. N = 369.

Primary Psychiatric Diagnosis	N (%)
Depression	176 (47.70)
Attention-Deficit Hyperactive Disorder (ADHD)	78 (21.14)
Bipolar	35 (9.49)
Anxiety	35 (9.49)
Other	23 (6.23)
Post-traumatic Stress Disorder (PTSD)	22 (5.96)
Psychotic Disorder	0

Overall, 208 (56.34%) adolescents presented with multiple previously diagnosed psychiatric disorders. Approximately half of the adolescents endorsed a history of aggressive behaviors (n = 303, 51.10%). The most common drug used among this cohort was marijuana (n = 257, 43.12%), followed by tobacco (n = 191, 32.05%). Over half of the adolescents in the study reported being currently sexually active (n = 343, 57.45%), with a mean age of first sexual encounter of 13.57 (SD = 2.62). Statistically significant associations were found between the presence of at least one psychiatric diagnosis and suicide attempts (p < 0.001), aggressive behavior (p = 0.018), traumatic experiences (p < 0.001), tobacco use (p < 0.001), and illicit drug use (p = 0.024) (Table 3). There was no statistically significant association between the presence of at least one psychiatric diagnosis and alcohol use or risky sexual behavior.

**Table 3:** The Relationship Between Biopsychosocial Variables and Psychiatric Diagnoses Among Homeless Adolescents. Due to the varying data availability of each record, the number of included records in each analysis has been included after each category name. ^a^p-Values indicate potential differences between those with at least one psychiatric diagnosis and those without. An unpaired t-test was used for age of sexual activity initiation while a chi-square test of independence was calculated for all other variables. Significant p-values are displayed in bold.

Biopsychosocial Factors	Entire Sample (N = 602)	Presence of Psychiatric Diagnosis (N = 369)	No Psychiatric Diagnosis (N = 229)	p-Value^a^
Behavioral History n (%)				
Suicide Attempt in the Past 6 Months (N = 594)	140 (23.57)	103 (27.91)	37 (16.16)	<0.001
Suicide Attempt Before Past 6 Months (N = 593)	245 (41.32)	189 (51.22)	56 (24.45)	<0.001
Aggressive Behavior in the Past 6 Months (N = 594)	210 (35.35)	143 (38.75)	67 (29.26)	0.018
Aggressive Behavior Before Past 6 Months (N = 593)	303 (51.10)	201 (54.47)	102 (44.54)	0.018
History of Traumatic Event(s) (N = 596)	198 (33.22)	143 (38.75)	55 (24.02)	<0.001
Social History n (%)				
Tobacco (N = 596)	191 (32.05)	139 (37.67)	52 (22.71)	<0.001
Alcohol (N = 595)	97 (16.30)	67 (18.16)	30 (13.10)	0.103
Marijuana (N = 596)	257 (43.12)	168 (45.53)	89 (38.86)	0.110
Other Drug(s) (N = 596)	54 (9.06)	41 (11.11)	13 (5.68)	0.024
Sexual History n (%)				
Sexually Active (N = 597)	343 (57.45)	219 (59.35)	124 (54.15)	0.211
Age Sexually Active Mean (SD) (N = 330)	13.57 (2.62)	13.45 (2.81)	13.78 (2.23)	0.279
Birth Control Use (N = 584)	216 (36.99)	137 (37.13)	79 (34.50)	0.515
History of Sexually Transmitted Infection (STI) (N = 587)	37 (6.30)	23 (6.23)	14 (6.11)	0.953

## Discussion

This single-site analysis found that the presence of at least one psychiatric diagnosis is correlated with previous suicide attempts, aggressive behavior, traumatic experiences, tobacco use, and illicit drug use. It also further illuminates the burden of psychiatric disease on the homeless youth population in an urban area, with 61.3% of adolescents currently suffering from at least one psychiatric illness. A previous interim analysis of this same population found that 54.9% of youths had been diagnosed with a psychiatric illness [4]. The findings presented in this final analysis represent a relatively recent 6.4% increase in disease prevalence whose cause remains unknown.

It is also important to consider that this population may be underdiagnosed as well due to more limited healthcare access in comparison to their housed peers. Previous studies have shown that homeless youth are more likely to experience trauma due to their disposition, substance use to cope with their social situations, and mental illnesses in adulthood due to early life adversity [5]. In addition to these factors, disadvantaged youth are less likely to report traumatic events and stressors, as they may fear the repercussions resulting from that reporting. This single-site study suggests that homeless youth with existing psychiatric pathology are potentially even more vulnerable to adversity resulting from their housing insecurity and psychiatric diagnoses.

An interim analysis of this population also showed that a history of psychiatric diagnoses was not significantly associated with any illicit drug use [4]. However, with the larger sample size presented here, a significant association arose between psychiatric diagnoses and either tobacco use or the use of drugs other than alcohol, tobacco, or marijuana (i.e., stimulants, opioids, etc.). Overall, homeless adolescents have been found to be two or three times more likely to abuse drugs than their housed counterparts, with rates of amphetamine and cocaine use about three to five times higher [6]. Our findings align with those presented by Gomez et al. in their 2010 paper, though the documentation used for this study did not provide specific drug types aside from alcohol, tobacco, and marijuana.

Adolescent suicidality, a major public health issue, is even more prevalent among this population [3]. Suicide is the leading cause of death among youths experiencing homelessness, even more so than homeless adults, despite the latter population being better understood and more thoroughly researched [7]. Among the youths included in this study, the presence of any existing psychiatric diagnosis was correlated with a history of suicide attempts. Though our results agree with previous research, this study most likely underestimates adolescent suicidality in the homeless youth population, as we were unable to track suicide mortality.

Throughout the past two decades, more resources have been directed toward interventions for homeless youth. A study assessing the impact of formal cognitive training among homeless adolescents demonstrated significantly increased cognitive performance and less psychological distress in the group that received the intervention [8]. However, during the implementation of the intervention, the study team saw a high drop-out rate among participants, likely due to the mercurial circumstances in which they live [8-10]. The problems encountered by Medalia et al. are hardly unique when attempting to implement longitudinal interventions among homeless adolescents because the study population is constantly in flux.

Such studies, in addition to our own, indicate that interventions geared at mental health promotion to counterbalance their circumstances in homeless adolescents should be considered. Crucial focus should be placed on developing methods to assist adolescents younger than 18 years old and higher-risk groups such as those with established psychiatric diagnoses. Because all psychiatric diagnoses are distinct from one another, it may be useful to perform further analysis to assess correlates of individual psychiatric diagnoses, such as depression, attention-deficit hyperactive disorder (ADHD), bipolar disorder, and schizoaffective disorder. This could provide a deeper understanding and clarify trends among specific psychiatric diagnoses of the adolescent homeless population.

Our study was limited by its status as a retrospective chart review, where data were gathered for clinical, rather than research, purposes. Due to the self-reported nature of the data, the study is also susceptible to recall bias and social desirability bias. Because the study was performed only at a single site, the data may not be generalizable or representative of the homeless adolescent population outside of the southeastern Pennsylvania area. In addition, there may be homeless youth in this area who did not receive services at Valley Youth House and who could not be represented in our study. We were also limited in our demographic data, with information gathered on gender but none related to sexual orientation. The sample size of transgender adolescents was too small to conduct meaningful analyses, not allowing us to fully assess the relationship between transgender identity and psychiatric diagnoses. Asian and Native American/Alaskan individuals were also not well-represented in the sample, so these groups could not be included in the statistical analyses.

Future studies should include multiple shelters for homeless youth in the analysis, as well as a better demographic representation of this specific population. Substance use, especially illicit substance use, among homeless adolescents with psychiatric pathology may be a meaningful point of future intervention. However, as previously noted, many of these individuals engage in substance use to cope with their social situations. Interventions aimed toward mental health promotion and improvement of their living and social situations should be undertaken first to make a substance use intervention more meaningful. Future interventions should be applied to all homeless adolescents, regardless of current social history, to improve their psychological well-being, further address removing barriers, and maintain interest in such programs among homeless youth, in order to address the high drop-out rate most studies see when dealing with this population.

## Conclusions

Because adolescents are at a critical point between childhood and adulthood where they are first establishing their autonomy, it is important to provide them with the tools to allow them to take ownership of their healthcare. This is complicated for our population at Valley Youth House, who is currently undergoing this transitional period in an unstable environment. By better assessing and characterizing this population, we are providing a greater base of knowledge from which future interventional studies may be designed.
